# Whole-genome sequence analysis of a clinical hypervirulent *Klebsiella variicola* strain recovered from a hospital in Ningxia, China

**DOI:** 10.3389/fmicb.2026.1961169

**Published:** 2026-10-07

**Authors:** Yuting Kang, Zezhou Zhang, Siyu Ma, Wei Tao, Pengtao Wang

**Affiliations:** 1 Ningxia Key Laboratory of Clinical and Pathogenic Microbiology, Institute of Medical Sciences, General Hospital of Ningxia Medical University, Yinchuan, Ningxia, China; 2 Department of Radiology, General Hospital of Ningxia Medical University, Yinchuan, Ningxia, China; 3 First Clinical Medical College, Ningxia Medical University, Yinchuan, Ningxia, China; 4 Department of Gastroenterology, General Hospital of Ningxia Medical University, Yinchuan, Ningxia, China

**Keywords:** antibiotic resistance, genetic analysis, *Klebsiella variicola*, virulence factors, whole-genome sequencing

## Abstract

*Klebsiella variicola* (KV) infections are being increasingly reported across diverse geographic regions and linked to high mortality. KV displays substantial geographical heterogeneity in antimicrobial resistance profiles, virulence phenotypes, and genetic backgrounds; genetic variants further promote its adaptability to varied environments. In this study, we isolated a strain designated KV7 from the sputum sample of an ICU patient at a tertiary hospital in Ningxia, China, with species confirmation achieved via whole-genome sequencing. Antimicrobial susceptibility testing was performed using the VITEK 2 Compact automated microbial system. String test, immune cell phagocytosis assay, serum resistance assay, and mouse infection model were applied to evaluate virulence phenotypes. Genomic characterization revealed that KV7 belongs to ST8223 with an unassigned KL type and exhibits hypervirulence while remaining susceptible to tested antibiotics. Its hypervirulence is associated with the virulence plasmid pKV7-vir, which carries numerous virulence factor genes. Multiple mobile genetic elements were also detected on pKV7-vir. Moreover, comparative genomic analysis of KV isolates from China and other countries was performed, laying a theoretical foundation for regional surveillance of this pathogen.

## 1 Introduction

*Klebsiella variicola* (KV) is a Gram-negative, facultatively anaerobic, non-spore-forming, non-motile rod bacterium that forms round, raised, and smooth colonies, capable of colonizing various hosts including plants, humans, insects, and animals (Rodríguez-Medina et al., 2019; Barrios-Camacho et al., 2019; Qin et al., 2023; Duran-Bedolla et al., 2021). Initially identified as an endophyte in plants, KV benefits plant growth and nutrient acquisition via nitrogen fixation (Duran-Bedolla et al., 2021). This bacterium is also widely distributed in diverse environmental niches such as contaminated soil, river water, and wastewater, and frequently establishes symbiotic associations with other bacterial species (Rodrigues et al., 2018; Nikapitiya et al., 2023; Wang D. et al., 2022; Gomi et al., 2018). Although KV primarily resides in environmental and plant-associated habitats, it functions as an opportunistic pathogen that can infect humans and animals (Rodríguez-Medina et al., 2019; Ohno et al., 2025). It has been implicated in bovine mastitis and infections in dogs, birds, and primates (Podder et al., 2014; Giannattasio-Ferraz et al., 2022; Brisse and Duijkeren, 2005). Clinically, KV has been isolated from multiple human specimens, including blood, tracheal aspirates, and various body secretions, and is responsible for respiratory tract infections, urinary tract infections, and surgical site infections (Rosenblueth et al., 2004; Ohama et al., 2022; Rodríguez-Medina et al., 2019). Accumulating clinical evidence has further confirmed the association between KV and bloodstream infections (BSIs), and KV-induced BSIs are associated with a high mortality rate in affected patients (Berry et al., 2015; Maatallah et al., 2014; Ota et al., 2026). In addition to its pathogenic potential, the extensive antimicrobial resistance (AMR) of KV poses a severe clinical challenge. A previous analysis of 28 clinical KV isolates revealed that nearly half of the tested strains produce broad-spectrum β-lactamases and carbapenemases, conferring resistance to first-line antimicrobial agents (Barrios-Camacho et al., 2019). Consistently, a recent global epidemiological investigation revealed that approximately 50% of circulating KV strains exhibit an extended-spectrum β-lactamase (ESBL) phenotype (Holt et al., 2015). Accordingly, KV is regarded as a critical reservoir of antimicrobial resistance (AMR) genes, greatly complicating clinical antimicrobial therapy and disease management.

The high virulence characteristic of KV have increasingly been recognized as significant pathogenic factors leading to severe clinical diseases (Lee et al., 2025; Morales-León et al., 2021). KV possesses multiple intrinsic virulence determinants—including adhesive capability, anti-phagocytic activity, efflux pumps, iron acquisition systems, regulatory elements, secretion apparatuses, and serum resistance—that collectively underpin its pathogenic potential. Notably, the plasmid-borne *rmpA* gene, which modulates capsular polysaccharide synthesis, along with iron transport-related genes *iroB* and *iucA*, acts as core virulence markers of KV (Russo et al., 2018). The mucoid phenotype is widely acknowledged as a hallmark of hypervirulent KV strains. In 2020, a study reported a hypermucoviscous KV that harbors diverse virulence-associated systems, including the *kfuABC* iron transport system, enterobactin-encoding *entB*, and the type 3 fimbrial gene cluster *mrkABCDFHIJ* (Rodríguez-Medina et al., 2020). Another virulent KV strain, KV093, isolated in India, carries multiple virulence-associated operons and genes linked to capsule synthesis (*cpsABCD*), lipopolysaccharide (LPS) production, fimbrial adhesion (*mrk* and *fim* clusters), and iron acquisition systems and corresponding receptors (Srinivasan and Rajamohan, 2020). Moreover, the hypervirulent KV strain X39, isolated from the sputum of patients with community-acquired pneumonia, harbors abundant iron-associated virulence factors, including *iroN*, *aerobactin*, and *enterobactin*. Overall, the rising prevalence of hypermucoviscous KV strains poses considerable clinical risks and raises serious public health concerns (Guo et al., 2018).

KV, *Klebsiella pneumoniae* (KP), and *Klebsiella quasipneumoniae* (KQ) collectively constitute the *Klebsiella complex*. Under routine microbiological culture conditions, members of this complex exhibit highly convergent phenotypic and biochemical characteristics (Ohama et al., 2022; Ohno et al., 2025). The high genetic homology and overlapping phenotypic profiles among these closely related species make conventional biochemical assays inadequate for accurate species-level differentiation. Conventional matrix-assisted laser desorption/ionization time-of-flight mass spectrometry, a widely used identification tool, frequently misclassifies species within the *Klebsiella complex*, with KV commonly mistaken for KP in clinical practice (Berry et al., 2015; Martínez-Romero et al., 2018). Such diagnostic inaccuracies have substantially resulted in the underestimation of the true public health burden attributable to KV infections (Fontana et al., 2019). Whole-genome sequencing (WGS) has emerged as a robust and promising gold-standard approach for precise species discrimination within the *Klebsiella* genus (Long et al., 2017; Huang et al., 2021). WGS has been extensively implemented in clinical microbiology diagnostics for pathogen screening, persistent surveillance, outbreak tracing, and epidemiological source tracking of critical bacterial pathogens (Snitkin et al., 2012). Notably, this high-resolution molecular technique allows for the identification of *Klebsiella complex* members without the need for microbial culture. Currently, WGS is routinely applied in multiple regions to accurately identify clinical KV isolates, comprehensively profile their antimicrobial resistance and virulence traits, and provide solid theoretical evidence to support precise clinical diagnosis and targeted antimicrobial treatment.

In this study, we isolated a strain of KV designated as KV7 from the sputum of ICU patients at a tertiary hospital in the Ningxia region. The identification was conducted using MALDI-TOF and WGS. We analyzed the antibiotic resistance and virulence characteristics of KV7, focusing on its resistance genomic features, virulence factors, and mobile elements. Furthermore, comparative phylogenetic analysis was performed using *K. variicola* isolates from both China and other countries. Our results provide a valuable theoretical foundation for regional surveillance of *K. variicola* infections.

## 2 Methods and materials

### 2.1 Isolation and antibiotic susceptibility testing of KV7

*Klebsiella variicola* strain KV7 was isolated from sputum of a 48-year-old male ICU patient hospitalized at the General Hospital of Ningxia Medical University. Species identification and antimicrobial susceptibility testing were carried out on the VITEK-2 Compact automated microbial analyzer (BioMerieux, Paris, France). The resulting susceptibility data were interpreted according to CLSI M100-S26 guidelines. Ethical approval for this study was granted by the Ethics Committee of the General Hospital of Ningxia Medical University. Strain identification and susceptibility tests were performed with standardized protocols to satisfy ethical compliance.

### 2.2 String test

Bacterial strains were cultured on blood agar under aerobic incubation at 37 °C for 18 h. Individual colonies were harvested using an inoculating loop for string test detection of viscous filament formation. A filament longer than 5 mm represented a positive string test result, suggestive of hypervirulent phenotype. All experiments were performed in triplicate.

### 2.3 Serum resistance assay

The serum-resistance assay were performed with minor modifications to a previously reported protocol (Liu et al., 2019). In short, 250 μL of mid-logarithmic-phase bacterial suspension (1 × 10^6^ CFU/mL) was combined with 750 μL PBS-diluted 20% heat-inactivated human serum and then incubated at 37 °C. Samples were collected at 0, 1, 2 and 3 h, serially diluted and plated on LB agar for colony enumeration. The bacterial survival rate was defined as the percentage of viable colonies at each time-point relative to the initial CFU value obtained at 0 h. All experiments were performed in triplicate.

### 2.4 Immune cell phagocytosis experiment

Phagocytosis tests were performed with the mouse alveolar macrophage MH-S cell line. Bacteria were cultured overnight until reaching mid-log phase. Bacteria and MH-S cells were co-cultured at an MOI of 10:1 at 37 °C, with sample collection at 0, 1, 2 and 3 h. Serially diluted samples were spread on agar plates for viable colony counting. The killing percentage was determined by dividing the number of surviving colonies in the MH-S co-culture group by the initial CFU count at 0 h. All experiments were performed in triplicate.

### 2.5 Mouse intraperitoneal infection model

Animal experiments were strictly performed in accordance with the National Institutes of Health guidelines for laboratory animal welfare. The animal study protocol was approved by the Medical Science Research Ethics Committee of the General Hospital of Ningxia Medical University (approval number: KYLL-2026-0351, approved on March 12, 2026). Female ICR mice aged 6–7 weeks were purchased from ViewSolid Biotech (Beijing, China) and randomly assigned into four experimental groups: PBS, KV7, ATCC 700603, and NTUH-K2044, with 10 mice per group. Bacterial strains were cultured in LB broth to logarithmic phase, and each mouse was challenged by intraperitoneal injection with 5 × 10^7^ CFU bacterial suspension. The PBS group served as the blank negative control, *Klebsiella quasipneumoniae* (KQ) ATCC 700603 was set as the low-virulence control strain, and *Klebsiella pneumoniae* (KP) NTUH-K2044 was used as the hypervirulent positive control strain. Mortality of mice was recorded every 12 h after bacterial challenge up to 72 h. Kaplan–Meier survival analysis was performed, and the log-rank (Mantel–Cox) test was used to compare survival differences across groups. Statistical significance was set at *p* < 0.05. All animal assays were performed in three independent replicates.

### 2.6 Whole genome sequencing of KV7

Genomic DNA of KV7 was extracted and sequenced using combined DNB short-read (350 bp paired-end) and PacBio long-read (10 kb library) strategies at BGI (Shenzhen, China). Genomic DNA was ultrasonically sheared and purified to obtain 300–400 bp fragments. Qualified libraries validated by an Agilent 2,100 Bioanalyzer were sequenced via the combinatorial probe anchor synthesis (cPAS) approach. Raw PacBio reads were trimmed and filtered to remove low-quality sequences. K-mer analysis was performed to evaluate genomic characteristics prior to *de novo* genome assembly using SPAdes v3.11.0. Gene functional annotation was conducted using RAST v2.0 and Prokka v1.12.21. The circular plasmid map was visualized with GenomeVx, while plasmid comparative analysis and genomic linear alignment were performed using BRIG v0.95 and EasyFigure v2.2.3, respectively. The VRprofile 2.0 platform was used for plasmid incompatibility grouping and prediction of mobile genetic elements and antimicrobial resistance-associated genomic features.

### 2.7 Average nucleotide identity (ANI) and digital DNA–DNA hybridization (dDDH) analysis

For genomic species identification and comparative genomic analysis, ANI and dDDH values were calculated between strain KV7 and other *Klebsiella* strains. ANI analysis was performed using the web version of EzBioCloud ANI Calculator,^1^ while dDDH values were estimated via the Type Strain Genome Server (TYGS) web server. The standard species threshold values of 95% ANI and 70% dDDH were applied to determine genomic relatedness and taxonomic affiliation.

### 2.8 Multilocus sequence typing (MLST) and Klebsiella capsular locus (KL) typing of KV7

MLST and KL typing of KV7 were performed based on whole-genome sequencing data. The assembled genome was subjected to in silico MLST analysis to determine the sequence type (ST) in accordance with the official KP MLST scheme, which targets seven conserved housekeeping genes (*gapA, infB, mdh, pgi, phoE, rpoB,* and *tonB*). Allele profiles and ST assignment were confirmed by alignment against the curated PubMLST database.^2^ Capsular KL-typing was attempted using Kaptive against the KP KL reference database.^3^

### 2.9 Phylogenetic tree analysis of KV7 within the *Klebsiella* complex

Whole-genome sequences of KV, KP, and KQ strains were generated in this study or retrieved from the NCBI database. All genomes were structurally and functionally annotated using Prokka v1.14.5. Roary v3.13.0 was used to resolve interspecies relationships within the *Klebsiella* complex; the core genome was defined via pan-genome clustering using a 95% protein identity cutoff, a strategy ideal for comparisons among closely related *Klebsiella* species. Conserved core-genome single-nucleotide polymorphisms (SNPs) were extracted and multiple-sequence aligned for phylogenetic reconstruction. A maximum-likelihood phylogenetic tree was constructed using Molecular Evolutionary Genetics Analysis (MEGA) to assess the robustness of each branch. The final phylogenetic topology was visualized using FigTree v1.4.4.

### 2.10 Analysis of phylogenetic homology among KV strains

To further explore the intraspecific phylogenetic homology and population genetic structure of KV strains, core-genome SNPs were extracted from whole-genome sequences using kSNP4. Substantial interspecies divergence within the *Klebsiella* complex disrupts k-mer conservation and generates artifactual SNPs. For this reason, kSNP4 yields reliable results only for closely related conspecific isolates. Briefly, kSNP4 identifies SNPs based on conserved k-mer sequences without requiring a single reference genome, and core-genome SNPs shared across all strains were retained for downstream phylogenetic analysis. A maximum-likelihood phylogenetic tree was generated using IQ-TREE v2.2.0, and the resulting Newick-formatted tree file was imported into Interactive Tree Of Life (iTOL v6) for circular phylogenetic visualization. Strain metadata, including isolation year, geographical origin, host source, MLST, and *wzi*-based capsular typing, were annotated as concentric colored rings surrounding the tree.

### 2.11 Statistical analysis

GraphPad Prism 8.0 was utilised for all statistical processing. Quantitative data are shown as medians or mean ± SD. One-way ANOVA and subsequent Tukey multiple comparison tests were used to evaluate differences among groups, with *p* < 0.05 defined as the threshold for significant discrepancy.

## 3 Results

### 3.1 Genomic characteristics and functional analysis of KV7

We performed whole genome sequencing and assembly of KV7, which revealed one chromosome of 5,412,823 bp (KV7-C) and one plasmid of 217,996 bp (pKV7-vir). The average GC content of the genome is 58.41%, comprising 5,386 genes. This includes non-coding RNAs (ncRNAs), which consist of 87 copies of tRNA and 25 copies of rRNA (specifically, 9 copies of 5S rRNA, 8 copies of 16S rRNA, and 8 copies of 23S rRNA), as well as 44 copies of sRNA (Figures 1A,B). Additionally, there are 120 tandem repeat sequences and 4 CRISPRs (Clustered Regularly Interspaced Short Palindromic Repeats). Gene Ontology (GO) functional enrichment analysis showed that genes associated with biological processes were dominated by those involved in metabolic processes (1,793 genes). In the cellular component category, 918 genes were annotated to cellular anatomical entities. For molecular functions, catalytic activity-related genes constituted the largest proportion, with a total of 1,990 genes (Figure 1C). Moreover, Cluster of Orthologous Groups (COG) functional classification further verified that the KV7 genome harbors a high abundance of genes related to transcription, amino acid transport and metabolism, and carbohydrate transport and metabolism (Figure 1D).

**Figure 1:**
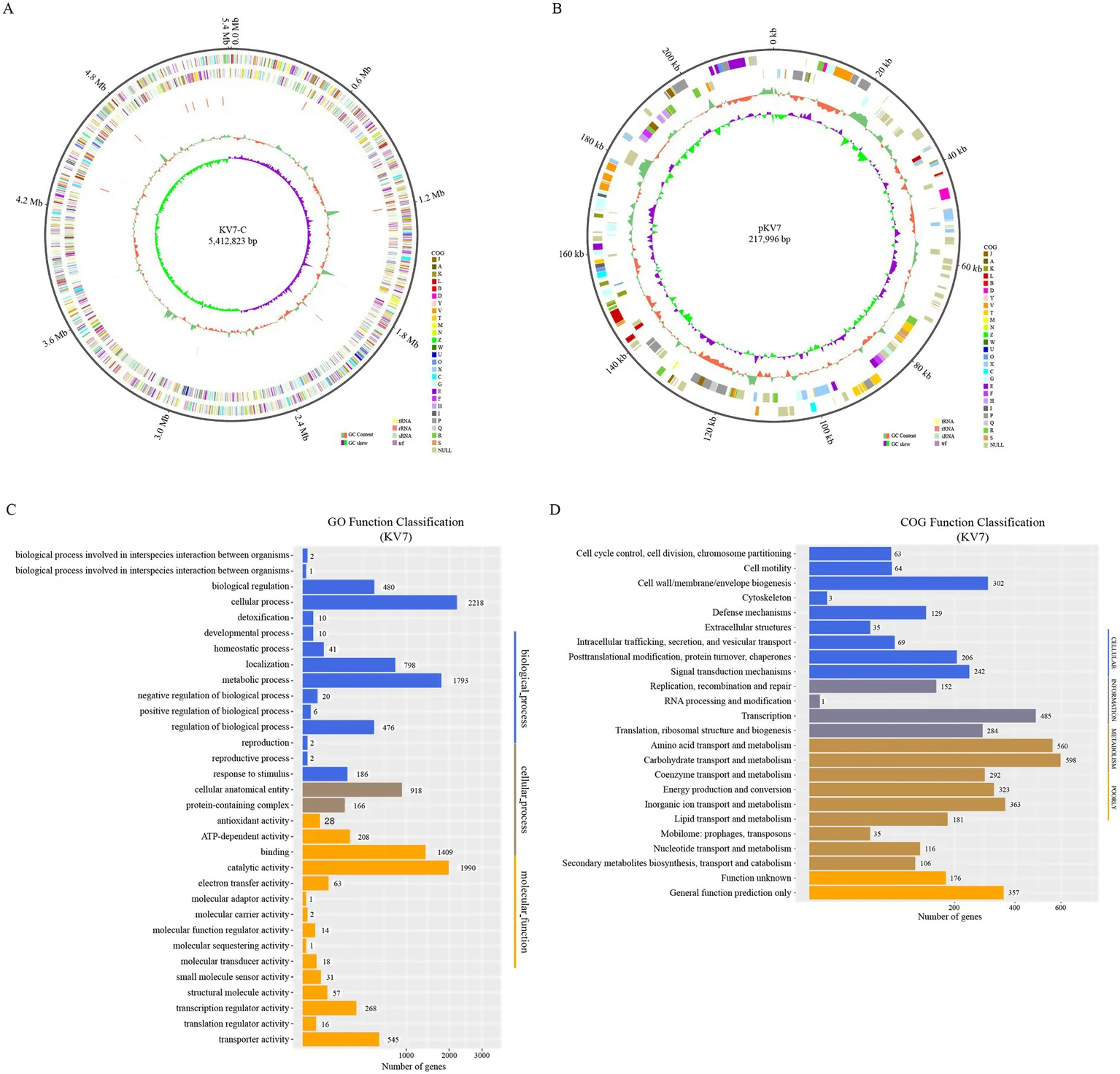
The genomic information characteristics of KV7 are presented as follows: **(A)** Chromosomal circular map, **(B)** Plasmid circular map, **(C)** Gene Ontology (GO) functional annotation, and **(D)** Kyoto Encyclopedia of Genes and Genomes (KEGG) functional annotation.

The antibiotic resistance and virulence genes carried by the chromosome and plasmid of KV7 were analyzed using the Comprehensive Antibiotic Resistance Database and Virulence Factors of Pathogenic Bacteria. The results showed that the chromosome harbors a significant number of resistance genes, including the β-lactamase resistance gene *bla*_LEN-24_, the efflux pump genes *kpnH*, *kpnG, kpnF, kpnE, CRP, baeR, marA, lptD, adeF, rsmA, msbA, H-NS,* the outer membrane gene *mdtQ*, the porin gene *ompK37*, the vancomycin resistance gene *vanG*, and *fosA6* (the chromosomally encoded fosfomycin resistance gene). The virulence genes carried by the chromosome of KV7 include Type I fimbriae genes *fimABCDEFGHIK*, Type 3 fimbriae genes *mrkABCDFHIJ*, Ent siderophore genes *entABCDEFS*, *fepABCDG*, salmochelin genes *iroEN*, and genes related to the T6SS system. The virulence plasmid pKV7-vir contains virulence factors such as *iucABCD, iutA*, and *iroBCDN*. All results are summarized and presented in Table 1.

**Table 1:** Resisitance and virulence genes harbored on the chromosome and plasmid of KV7.

Gene category	Chromosome(KV7-C)	Plasmid (pKV7-vir)
Resistance genes		
β-lactam	*bla*_LEN-24_, *CRP, kpnH, kpnG, mdtQ, H-NS, marA, ompK37*	-
Aminoglycoside	*kpnH, kpnG, kpnF, kpnE, baeR*	-
Carbapenem	*kpnH, kpnG, mdtQ, marA, OmpK37, lptD*	-
Fluoroquinolone	*CRP, rsmA, kpnH, kpnG, emrR, adeF, oqxA, adeF, H-NS, marA, adeF*	-
Glycopeptide	*vanG*	-
Macrolide	*kpnH, kpnG, kpnF, kpnE, H-NS, CRP,*	-
Monobactam	*mdtQ, marA, mmpK37,*	-
Nitroimidazole	*msbA*	-
Phosphonic acid	*fosA6*	-
Rifamycin	*marA, kpnF, kpnE, lptD*	-
Cephalosporin	*kpnH, kpnG, kpnF, kpnE, ompK37, mdtQ, H-NS, marA,*	-
Tetracycline	*adeF, oqxA, adeF, H-NS, marA, kpnF, kpnE, adeF*	-
Glycylcycline	*oqxA, marA*	-
Virulence genes		
Type 1 fimbriae	*fimABCDEFGHIK*	-
Type 3 fimbriae	*mrkABCDFHIJ*	-
AcrAB	*acrAB*	-
Aerobactin	*-*	iucABCD,iutA
Ent siderophore	*entABCDEFS, fepABCDG, fes*	-
Salmochelin	*iroEN*	iroBCDN
RcsAB	*rcsAB*	-
T6SS-I	*clpV/tssH, dotU/tssL, hcp/tssD, icmF/tssM, ompA, sciN/tssJ, tle1, tli1, tssF, tssG, vasE/tssK, vgrG/tssI, vipA/tssB, vipB/tssC*	-
T6SS-II	*clpV*	-
T6SS-III	*dotU, icmF, impA, impF, impG, impH, impJ, ompA, sciN, vgrG*	-
Salmonella fimbrial protein	*stcBC, stiB*	-
Mg2 + transport(Salmonella)	*mgtB*	-

### 3.2 Phylogenetic tree analysis of homology between KV7 and different members of the *Klebsiella* complex

We utilized the Basic Local Alignment Search Tool (BLAST) to assess the genome of isolate KV7 and employed Molecular Evolutionary Genetics Analysis (MEGA) software to construct a phylogenetic tree to further confirm the taxonomic homology of KV7. The results indicate that KV7 shares high homology with other KV strains, specifically KV342 (GenBank accession number CP000964.1), DX120E (GenBank accession number CP009274.2), At-22 (GenBank accession number CP001891.1), and X39 (GenBank accession number CP018307.1), while its homology with KP and KQ is relatively distant (Figure 2). KV7 exhibits the highest homology with KV342, showing over 99% identity and 96% coverage (Table 2). Average nucleotide identity (ANI) and digital DNA–DNA hybridization (dDDH) analyses were performed to further confirm the taxonomic status of strain KV7. The results showed that the ANI values between KV7 and previously reported KV strains were all higher than 95%, while the corresponding dDDH values exceeded 70% (Table 3). These genomic indexes fully met the authoritative species delineation criteria for prokaryotes, which further verified that strain KV7 belongs to *Klebsiella variicola*.

**Figure 2:**
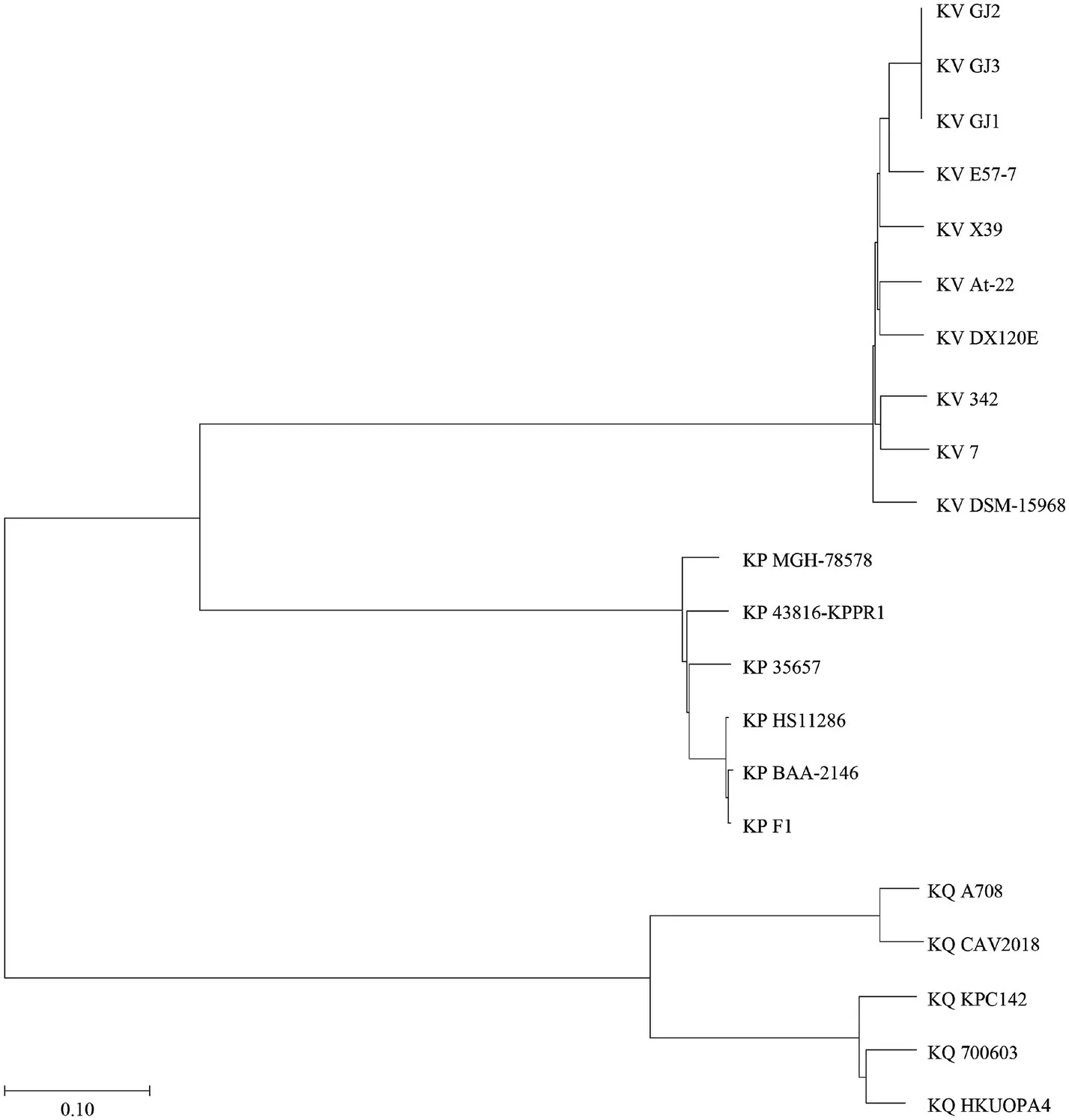
Phylogenetic tree analysis of KV7 within the Klebsiella complex was constructed. The strains are grouped into three major clusters: *Klebsiella variicola* (top branch, labeled KV), *Klebsiella pneumoniae* (middle branch, labeled KP), and *Klebsiella quasipneumoniae* (bottom branch, labeled KQ). The scale bar (0.10) indicates the genetic distance in terms of nucleotide substitutions per site.

**Table 2:** The information of the Klebsiella isolates used to construct phylogenetic tree.

Isolate name	capsular locus	ST	geographic origins	origin	Assession number	Coverage (%)	Identify (%)
*K. pneumoniae ATCC35657*	KL64	505	USA	unknown	CP015134.1	90	95.38
*K. pneumoniae F1*	KL47	11	China	urine	CP026130.1	85	95.37
*K. pneumoniae MGH-78578*	KL52	38	USA	sputum	CP000647.1	88	95.357
*K. pneumoniae ATCC 43816 KPPR1*	KL2	493	USA	Unknown	CP009208.1	88	95.32
*K. pneumoniae ATCC BAA-2146*	KL74	11	USA	urine	CP006659.2	87	95.37
*K. pneumoniae HS11286*	KL103	11	China	sputum	CP003200.1	87	95.38
*K. variicola 342*	KL30	146	USA	maize root	CP000964.1	95	99.507
*K. variicola At-22*	KL134	1,220	USA	*Atta cephalotes* fungus garden	CP001891.1	95	99.226
*K. variicola DX120E*	KL103	Not defined	China	sugarcane root	CP009274.2	95	99.342
*K. variicola DSM 15968*	KL16	2,263	China	banana root	CP010523.2	94	99.37
*K. variicola GJ1*	KL53	363	Korea	river	CP017284.1	94	99.533
*K. variicola GJ2*	KL53	363	Korea	river	CP017849.1	94	99.533
*K. variicola GJ3*	KL53	363	Korea	river	CP017289.1	94	99.533
*K. variicola* E57-7	KL103	697	China	maize root	CP016344.1	94	99.494
*K. variicola X39*	Not defined	718	China	Human (respiratory)	CP018307.1	94	99.481
*K. quasipneumoniae* ATCC 700603	KL53	489	USA	Human	CP014696.2	90	94.358
*K. quasipneumoniae* A708	KL23	Not defined	China	Human	CP030171.1	89	95.093
*K. quasipneumoniae* CAV2018	KL46	196	UK	Hospital waste water	CP029432.1	89	95.087
*K. quasipneumoniae* HKUOPA4	KL8	Not defined	China	giant panda feces	CP014154.1	88	94.332
*K. quasipneumoniae* KPC142	KL16	Not defined	Brazil	Human (respiratory)	CP023478.1	88	94.101

**Table 3:** The ANI and dDDH analysis between KV7 and other *Klebsiella* strains were performed.

Isolate name	ANI (%)	dDDH (%)
*K. pneumoniae ATCC35657*	94.61	58.5
*K. pneumoniae F1*	94.68	58.5
*K. pneumoniae MGH-78578*	94.67	58.6
*K. pneumoniae ATCC 43816 KPPR1*	94.66	58.8
*K. pneumoniae ATCC BAA-2146*	94.60	58.1
*K. pneumoniae HS11286*	94.69	58.3
*K. variicola 342*	99.04	92.4
*K. variicola At-22*	99.14	92.9
*K. variicola DX120E*	99.06	92.2
*K. variicola DSM 15968*	99.08	92.7
*K. variicola GJ1*	99.10	92.7
*K. variicola GJ2*	99.09	92.7
*K. variicola GJ3*	99.08	92.7
*K. variicola* E57-7	99.12	92.6
*K. variicola X39*	99.08	92.1
*K. quasipneumoniae* ATCC 700603	93.63	53.0
*K. quasipneumoniae* A708	93.49	52.7
*K. quasipneumoniae* CAV2018	93.46	52.7
*K. quasipneumoniae* HKUOPA4	93.54	53.2
*K. quasipneumoniae* KPC142	93.55	53.1

### 3.3 Antimicrobial resistance and virulence phenotype of KV7

Genomic analysis of KV7 indicates that the chromosome carries a significant number of resistance genes; however, no resistance plasmids were detected. Antimicrobial susceptibility testing indicates that KV7 is sensitive to all 20 antibiotics tested, including penicillins, β-lactamase inhibitors, cephalosporins, carbapenems, aminoglycosides, fluoroquinolones, nitrofurans, and sulfonamides (Table 4). The string test results for KV7 were positive, indicating a high level of mucid production, akin to the highly virulent strain NTUH-2044 (Figure 3A). In the mouse infection model, the 7-day survival rate of mice infected with the KV7 strain was 30%, which was slightly higher than the 20% survival rate observed for the hypervirulent strain NTUH-K2044. However, both values were significantly lower than that of the low-virulence reference strain KQ ATCC 700603 (a closely related *Klebsiella* complex species), which exhibited a 90% mouse survival rate at 7 days post-infection (Figure 3B). Serum resistance assays showed that both KV7 and NTUH-2044 displayed strong serum resistance levels compared to ATCC 700603 (Figure 3C). Additionally, macrophage phagocytosis assays indicated that after 2 to 3 h of interaction between the bacteria and cells, KV7 exhibited a significantly higher survival rate compared to ATCC 700603 (Figure 3D). These *in vitro* and *in vivo* experimental results collectively demonstrate that KV7 is a highly virulent strain.

**Table 4:** The antimicrobial susceptibility profile of KV7.

Antimicrobial agent	MIC(ug/ml)
Ampicillin/Sulbactam	≤2(S)
Piperacillin	≤4(S)
Piperacillin/Tazobactam	≤4(S)
Cefazolin	≤4(S)
Cefuroxime	≤1(S)
Cefuroxime Axetil	≤1(S)
Ceftotaxime	≤4(S)
Ceftazidime	≤1(S)
Ceftriaxone	≤1(S)
Cefepime	≤1(S)
Aztreonam	≤1(S)
Imipenem	≤1(S)
Meropenem	≤0.25(S)
Amikacin	≤2(S)
Gentamicin	≤1(S)
Tobramycin	≤1(S)
Ciprofloxacin	≤0.25(S)
Levofloxacin	≤0.25(S)
Nitrofurantoin	32(S)
Trimethoprim/Sulfamethoxazole	≤20(S)

**Figure 3:**
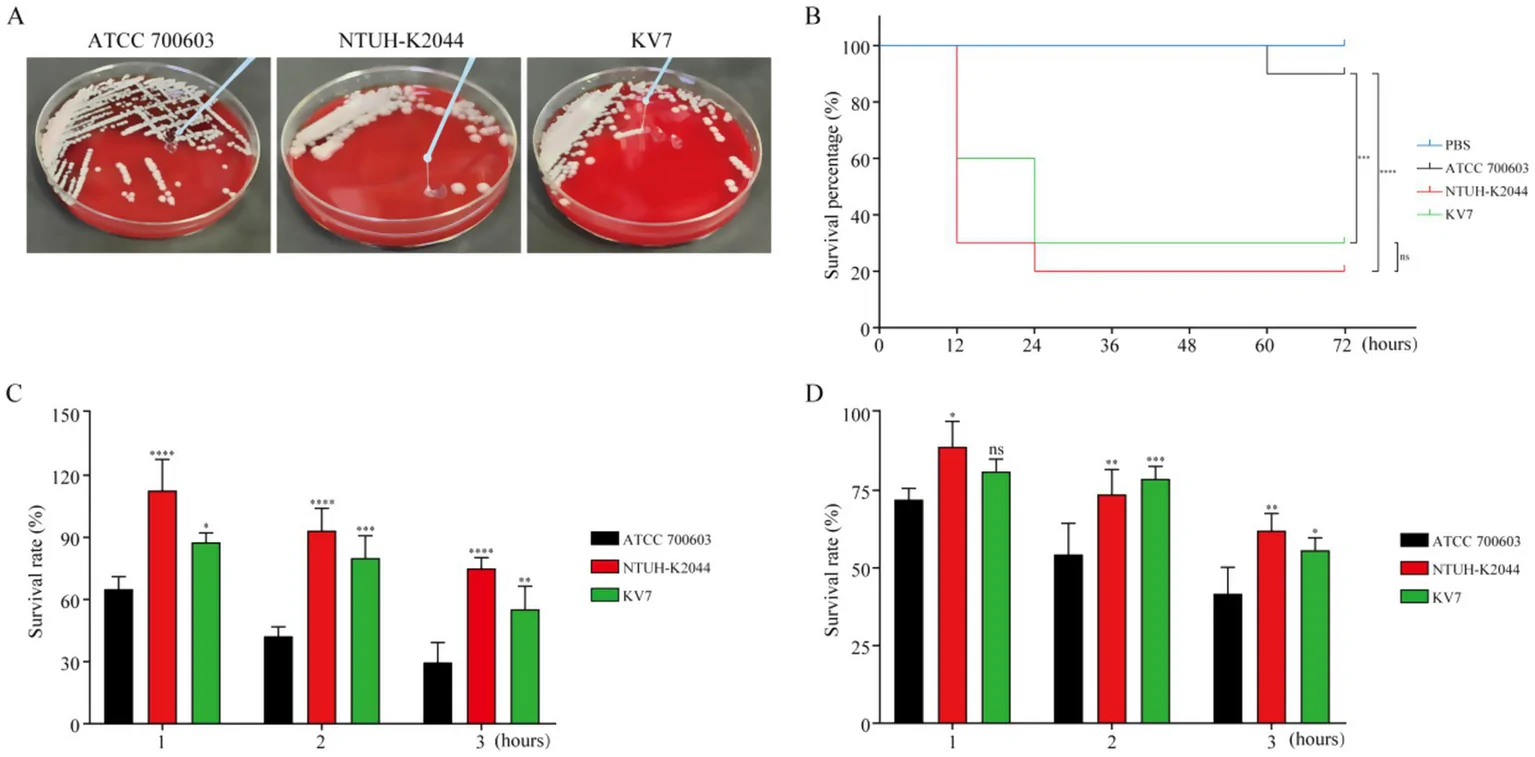
Virulence characteristics of the KV7 strain were evaluated via four experimental assays, including **(A)** string test, **(B)** mouse infection assay, **(C)** serum resistance assay, and **(D)** macrophage phagocytosis assay. The low-virulence reference strain KQ ATCC 700603 and hypervirulent reference strain KP NTUH-K2044 were used as the low-virulence and hypervirulent control groups, respectively. Statistical significance: ns, no significant difference; *p < 0.05, **p < 0.01, ***p < 0.001, ****p < 0.0001.

### 3.4 Comparative analysis of the pKV7-vir

We analyzed the high virulence characteristics of KV7 through both *in vitro* and *in vivo* experiments, which may be related to the virulence plasmid pKV7-vir carried by KV7. Therefore, we conducted a comparative analysis with other reported virulence plasmids associated with *Klebsiella* species (p15WZ-82_Vir, pLAhvKp217, pLVPK, pK2044, and pVir-CR-HvKP4). The results showed that pKV7-vir had over 99% identity with these plasmids, with the highest coverage of 83% when compared to pLAhvKp217 from KP, 57% coverage with p15WZ-82_Vir from KV, and 76 and 77% coverage with the classical virulence plasmids pLVPK and pK2044, respectively. The pKV7-vir plasmid carries two virulence gene regions: the iroBCDN and rmpA located in the 16–20 kb region, and the iucABCD and iutA located in the 120–128 kb region. These virulence regions are also present in the plasmids p15WZ-82_Vir, pLAhvKp217, pLVPK, and pK2044 (Figure 4A).

**Figure 4:**
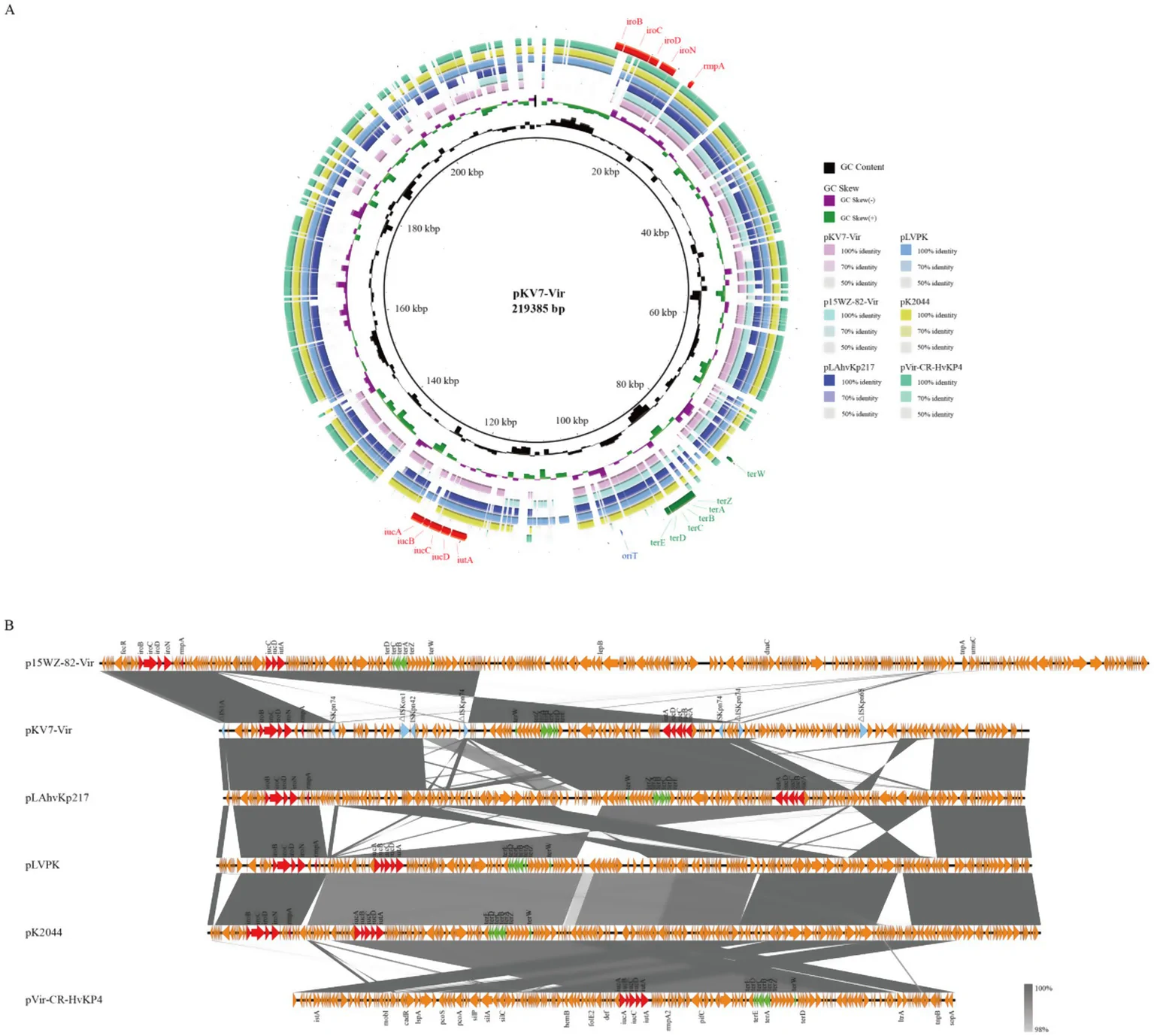
A comparative analysis of virulence factors and mobile elements between the virulence plasmid of KV7 (pKV7-vir) and other known virulence plasmids, including p15WZ-82_Vir, pLAhvKp217, pLVPK, pK2044, and pVir-CR-HvKP4, is presented. **(A)** A circle map analysis of the virulence factors present on these plasmids is provided. **(B)** A linear comparative analysis of the virulence factors and mobile elements across the plasmids is included.

Linear comparative genomic analysis revealed that pKV7-vir carries the tellurium resistance gene cluster *terZABCDW*. This operon improves bacterial fitness and survival under macrophage assault, and the *ter* operon harbored by hypervirulence plasmids has a significant association with *Klebsiella pneumoniae* infections (Yang et al., 2025). These conserved *ter* genes are present in other virulence plasmids we compared. In addition, various mobile genetic elements, including *ΔIS1A, ISKpn74, ΔISKpn74, ΔISKox1, ISKpn42,* and *ΔISKpn65*, are distributed in the upstream and downstream regions of the virulence factors of pKV7-vir, suggesting that they may facilitate the mobility of these virulence factors between species (Figure 4B).

### 3.5 Homology analysis of KV isolated from different regions

We conducted a phylogenetic tree analysis on a total of 166 KV strains reported in the literature and uploaded genomic sequences from 1991 to 2024. The results indicate that among the host sources, 117 strains are from humans, 20 from the environment, 16 from animals, 11 from plants, and 1 from an unknown source. The years of isolation are primarily concentrated between 2015 and 2019. MLST of the KV7 genome via the official Pasteur *Klebsiella* MLST database identified ST8223 (allelic profile: *gapA-426, infB-24, mdh-46, pgi-102, phoE-645, rpoB-22, tonB-178*). Kaptive-based KL typing against the KP reference database returned no valid KL assignment, likely due to marked sequence divergence of the KV capsule biosynthesis locus from KP reference sequences. Geographically, the majority of the strains are from Norway (69 strains) and China (46 strains). A total of 96 MLST types were identified, exhibiting a discrete distribution, with the highest proportions being ST347 with 10 strains, ST641 with 6 strains, and ST363 with 5 strains. The remaining ST types each had fewer than 5 strains, and there are 10 ST types that have not yet been identified, indicating a high degree of genetic variation in KV. Among the 34 strains identified as KL types, KL53 is the predominant type with 11 strains. Phylogenetic tree analysis shows that the KV7 and KV347 (GenBank accession number CP078755) strains form two independent branches, which are distantly related to the other strains. Despite the isolation of many ST and KL types, there are no prevalent ST and KL types in KV within each region.

The absence of geographically widespread, dominant ST clones suggests that human KV infections arise predominantly from independent, sporadic emergence of distinct lineages, rather than clonal dissemination of a single pandemic strain. The scattered distribution of STs, alongside the identification of 10 novel sequence types, reflects high standing genetic diversity within the species, consistent with a large, recombining bacterial population. Phylogenetically, KV7 (ST8223) and the ST347 reference strain CP078755 formed two deeply separated subclades distant from the majority of global KV isolates. This placement indicates ST8223 is a genetically distinct lineage within the global KV population. Although it lacks acquired resistance plasmids, KV7 exhibited high virulence *in vitro* and *in vivo*. Even with diverse KL capsular types observed, no dominant capsule lineage was enriched in particular hosts or regions. Unlike pandemic high-risk clones of KP, the KV population appears to be structured by recombination and strain dispersal, instead of clonal sweeps (Figure 5).

**Figure 5:**
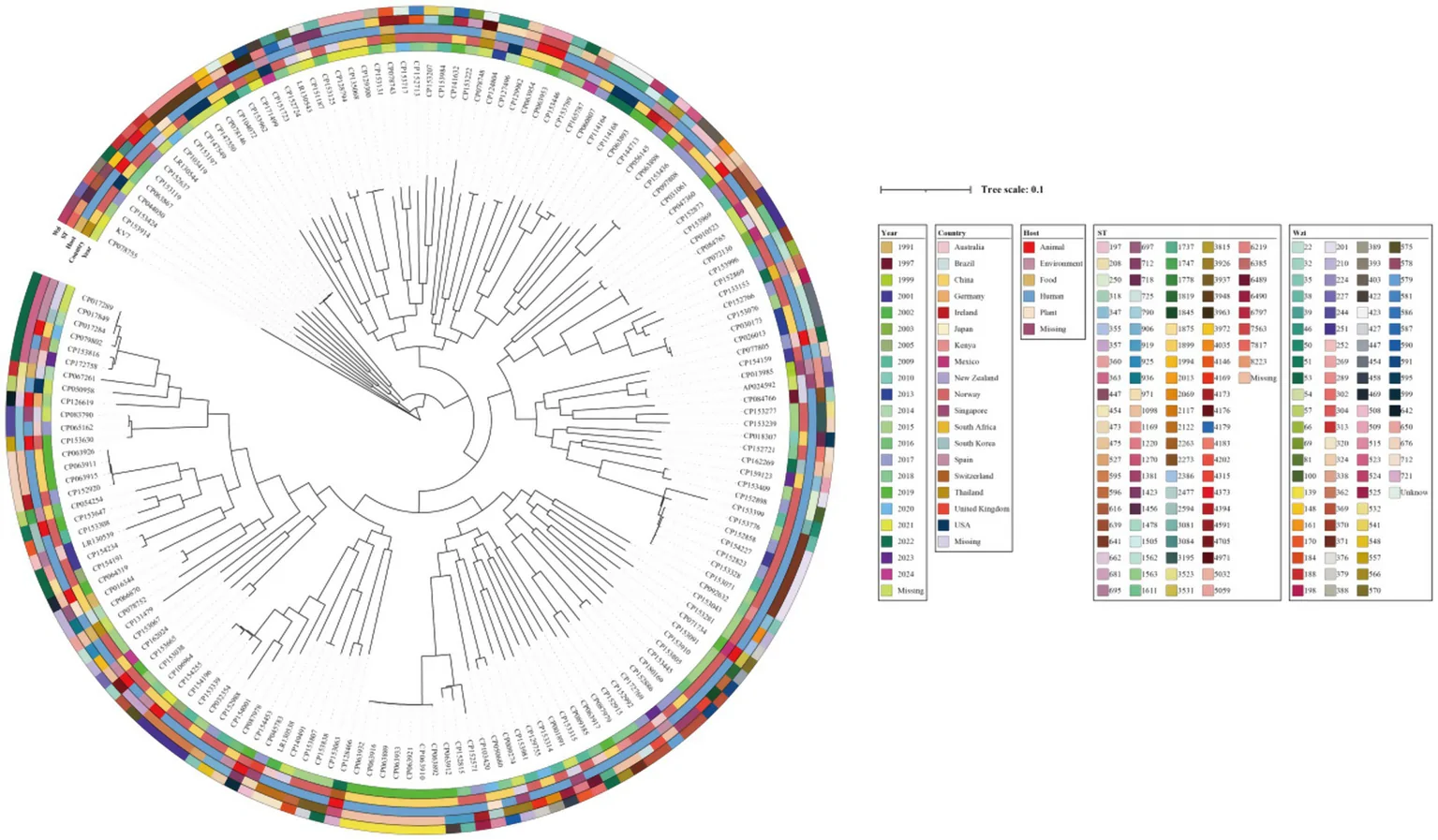
A circular maximum-likelihood phylogenetic tree of KV strains was constructed based on core-genome SNPs detected by kSNP4 and visualized using iTOL in a circular layout. Concentric color strips from the inner to outer ring represent isolation year, country of origin, host source, MLST, and wzi gene-derived capsular typing, respectively. The scale bar indicates genetic distance.

## 4 Discussion

*Klebsiella variicola* (KV) is an emerging human pathogen that can be isolated from diverse environmental niches. Similar to *Klebsiella pneumoniae* (KP), KV functions as an opportunistic pathogen and is capable of causing a spectrum of invasive infections, including bloodstream infections (BSIs), respiratory tract infections, and urinary tract infections (UTIs)(Holt et al., 2015; Imai et al., 2019; Rodríguez-Medina et al., 2019). A previous clinical study demonstrated that patients with KV-associated BSIs exhibit a markedly higher 30-day mortality rate (29.4%) than those infected with KP (13.5%) or *Klebsiella quasipneumoniae* (KQ) (11.1%) (Maatallah et al., 2014). Accordingly, clinical attention to KV-mediated infections has grown substantially in recent years. The enhanced pathogenic potential of KV is tightly linked to the carriage of virulence-associated genes, such as *peg-344*, *iucA*, and *rmpA*. The emergence of hypervirulent KV strains can induce severe clinical complications, posing a growing public health threat (Farzana et al., 2019; Ohno et al., 2025). Here, we firstly isolated a highly virulent KV strain, designated KV7, from a tertiary hospital in Ningxia, northwest China. This strain exhibits strong mucosity, serum resistance, and resistance to macrophage phagocytosis, and animal infection assays confirmed its high lethal potency in mice. Whole-genome sequencing analysis further uncovered abundant virulence factors and mobile genetic elements distributed across the chromosome and virulence plasmid of the KV7 strain. Phylogenetic analysis revealed that the KL serotype of the KV7 strain belongs to an unreported genotype, indicating that KV7 represents a novel KV clone. Given that the current KL databases lack sufficient reference sequences for KV capsule loci, no reliable matched KL type could be assigned to the KV7 isolate. Collectively, these findings highlight the emergence of a novel pathogenic KV clone, which necessitates sustained epidemiological surveillance to block its transmission and prevent potential outbreaks.

Multidrug-resistant KV strains have been documented in China, Japan, the United States, and Europe (Gu et al., 2018; Barrios-Camacho et al., 2019). Notably, the newly isolated KV7 strain in this study was susceptible to all 20 antibiotics tested and carried no resistance plasmids. This phenotypic profile is consistent with five KV strains recovered in Japan, which lacked extended-spectrum β-lactamase (ESBL) genes and displayed universal susceptibility to nearly all antimicrobial agents (Matsuda et al., 2023). Consistent with these findings, a recent South Korean study demonstrated that most clinical KV isolates (89.5%, 68/76) were susceptible to all 18 tested antimicrobials, indicating an overall low antibiotic resistance rate among circulating KV strains (Lee et al., 2025). Despite the absence of resistance plasmids in KV7, its chromosome encodes multiple β-lactamase and efflux pump genes, which may facilitate the development of antibiotic resistance. Similarly, the previously reported KV strain UFMG-H9, despite harboring no resistance plasmids, carries a variety of chromosomal resistance genes responsible for resistance to β-lactams (*bla*_LEN-16_, *bla*_LEN-19_), fosfomycin (*fosA*), and quinolones (*oqxA, oqxB*), and exhibits phenotypic resistance to ampicillin, erythromycin, and vancomycin (Giannattasio-Ferraz et al., 2022). Chromosomally encoded resistance genes act as a latent reservoir of MDR determinants. These genetic elements can drive intra- and interspecific gene dissemination, especially under clinical antibiotic selective pressure, ultimately promoting the emergence and spread of MDR KV clones.

We confirmed through both *in vivo* and *in vitro* experiments that the KV7 strain belongs to a high-virulence strain. Genetic analysis confirmed that KV7 carries a virulence plasmid and includes traditional virulence factors such as *rmpA* (which regulates the production of capsular polysaccharides that increase mucous viscosity), iron transport-related genes from the *iro* family, and *iuc* family genes (Matsuda et al., 2023; Yang et al., 2026). These virulence determinants have been well characterized in clinical isolates of KP and KV (Lu et al., 2018; Wang et al., 2024). However, a previous study reported that the hypermucoviscous phenotype of certain KV isolates can be mediated via *rmpA*-independent mechanisms (Ohno et al., 2025). In this study, the virulence-factor-enriched KV7 strain exhibited high lethality in mouse infection models, with a mortality rate comparable to that of the classic hypervirulent KP strain NTUH-K2044. Moreover, KV7 displayed serum resistance and anti-phagocytic capacity against macrophages equivalent to NTUH-K2044, and significantly higher than those of the low-virulence KQ strain ATCC 700603. Hypervirulent KV strains have been linked to severe invasive infections worldwide. Japanese studies have documented two cases of community-acquired liver abscesses caused by hypervirulent KV (Ohno et al., 2025). In Bangladesh, an outbreak of neonatal sepsis mediated by multidrug-resistant and hypervirulent KV was reported, with a notably high mortality rate (Farzana et al., 2019). Notably, KV7 harbors the tellurium resistance gene cluster *terZABCDW*. This gene cluster not only confers high-level tellurium resistance but also enhances the environmental adaptability and colonization capacity of the strain, thereby providing a competitive survival advantage (Yang et al., 2025; Turkovicova et al., 2016). The *ter* cluster is tightly associated with hypervirulent plasmids in KP, and clinical investigations have verified a significant correlation between ter genes and KP infectious diseases (Vornhagen et al., 2021; Passet and Brisse, 2015). Moreover, classical virulence plasmids, including p15WZ-82_Vir, pLAhvKp217, pLVPK, and pK2044, all carry conserved *ter* genes. The broad distribution of these virulence determinants across various plasmids is driven by abundant mobile genetic elements. Specifically, the ISKpn74 element is prevalent in most ST23-KL1 KP strains, indicating that such insertion elements substantially promote the horizontal transmission of virulence genes (Wang S. et al., 2022). In addition to virulence plasmids, the chromosome of KV7 encodes a diverse array of virulence-related determinants, including fimbrial genes and the type VI secretion system (T6SS). These genetic elements have been reported to potentiate KV pathogenicity and govern multiple pivotal bacterial phenotypic traits, including adhesion, motility, and host colonization capacity (Lane et al., 2007; Giannattasio-Ferraz et al., 2022).

Although the KV7 strains we isolated are from clinical settings, KV is widely distributed across plants, animals, and various environments, reflecting its remarkable survival capabilities (Martin and Bachman, 2018; Barrios-Camacho et al., 2019). Consistent with prior investigations, KV strains are frequently recovered from a broad range of sources such as plants, animals, insects and environmental samples, among which human clinical specimens constitute the dominant reservoir (Duran-Bedolla et al., 2021; Barrios-Camacho et al., 2019). Phylogenetic analysis shows no clear genetic clustering or segregation by isolation source, and no strong association exists between MLST sequence types and KL capsular serotypes, further illustrating the substantial phylogenetic diversity within KV (Duran-Bedolla et al., 2021). This pattern suggests frequent cross-regional dissemination of KV, rather than stable, geographically confined endemic clones. The scattered distribution of STs, alongside the identification of 10 novel sequence types, reflects high standing genetic diversity within the species, consistent with a large, recombining bacterial population. Beyond human infections, KV also causes multiple animal diseases, including bovine mastitis and invasive infections in dogs, birds, and primates (Podder et al., 2014; Brisse and Duijkeren, 2005). Epidemiological data demonstrate that some sequence types (STs) exhibit global distribution characteristics, and some isolates from different sources belong to the same ST, suggesting that KV can be transmitted across species (Barrios-Camacho et al., 2019). Although KV7 is classified as a sensitive strain, a large number of strains producing extended-spectrum β-lactamases (ESBLs) and carbapenemases have been identified globally (de Campos et al., 2021; Watanabe et al., 2026; Di et al., 2017). Among 50 strains of KV isolated from Hawassa Hospital that carry ESBL genes, both IncFIB (K) and IncFII (K) plasmids were detected (Legese et al., 2025). The presence of plasmids aids bacterial evolution, enabling adaptation to environments and conferring antibiotic resistance (Duran-Bedolla et al., 2023). Therefore, continuous clinical surveillance is critically required for the ST8223 KV7 strain, as it may acquire enhanced drug resistance in the future via spontaneous genetic mutations or horizontal transfer of exogenous resistance plasmids.

Nevertheless, the present study has several limitations that merit further exploration. First, only a single hypervirulent KV strain was isolated and identified from Ningxia region in this work. Therefore, we intend to screen more clinical KV isolates collected locally. An expanded strain library will facilitate subsequent investigations into the prevalence, clonal circulation, and transmission potential of hypervirulent clones and virulence plasmids in this region. Second, current data only establish a correlation between the carriage of the pKV7-vir plasmid and the hypervirulent phenotype of the KV7 strain, rather than confirming a definitive causal relationship. To resolve this limitation, follow-up experiments including plasmid curing, knockout of plasmid-encoded virulence genes, bacterial conjugation, and genetic complementation will be conducted. Third, the previously adopted low-virulence control strain ATCC 700603 belongs to KQ, while the present study focuses on the pathogenic characteristics of KV. The use of a heterologous *Klebsiella* species as a control may introduce experimental background interference and weaken the robustness of intraspecific virulence comparison. Accordingly, we will replace this heterologous strain with the canonical KV type strain DSM 15968 (ATCC BAA-830). This reference strain lacks hypervirulence plasmids and core virulence genes, making it an optimal baseline control for the pathogenicity analysis of KV.

## 5 Conclusion

This study firstly isolated and genetically characterized a clinically derived ST8223 *Klebsiella variicola* strain (KV7) with antibiotic susceptibility and hypervirulent phenotypes from a tertiary hospital in Ningxia, China. Genomic analysis demonstrated that the KV7 genome contains a virulence plasmid carrying numerous virulence-associated genes and mobile genetic elements. Given the detected virulence profiles of KV7 and the transmissible potential of its mobile genetic elements, sustained epidemiological monitoring is warranted. Targeted preventive measures should be considered to reduce the potential risk of nosocomial transmission and invasive infections caused by this emerging strain.

## Data Availability

The datasets presented in this study can be found in online repositories. The names of the repository/repositories and accession number(s) can be found at: https://www.ncbi.nlm.nih.gov/, CM127518.1.

## Footnotes

1
https://www.ezbiocloud.net/tools/ani

2
https://bigsdb.pasteur.fr/klebsiella/

3
https://kaptive-web.erc.monash.edu/
